# The POU Factor Ventral Veins Lacking/Drifter Directs the Timing of Metamorphosis through Ecdysteroid and Juvenile Hormone Signaling

**DOI:** 10.1371/journal.pgen.1004425

**Published:** 2014-06-19

**Authors:** CeCe Cheng, Amy Ko, Leila Chaieb, Takashi Koyama, Prioty Sarwar, Christen K. Mirth, Wendy A. Smith, Yuichiro Suzuki

**Affiliations:** 1 Department of Biological Sciences, Wellesley College, Wellesley, Massachusetts, United States of America; 2 Development, Evolution and the Environment Lab, Instituto Gulbenkian de Ciência, Oeiras, Portugal; 3 Department of Biology, Northeastern University, Boston, Massachusetts, United States of America; Stanford University School of Medicine, United States of America

## Abstract

Although endocrine changes are known to modulate the timing of major developmental transitions, the genetic mechanisms underlying these changes remain poorly understood. In insects, two developmental hormones, juvenile hormone (JH) and ecdysteroids, are coordinated with each other to induce developmental changes associated with metamorphosis. However, the regulation underlying the coordination of JH and ecdysteroid synthesis remains elusive. Here, we examined the function of a homolog of the vertebrate POU domain protein, Ventral veins lacking (Vvl)/Drifter, in regulating both of these hormonal pathways in the red flour beetle, *Tribolium castaneum* (Tenebrionidae). RNA interference-mediated silencing of *vvl* expression led to both precocious metamorphosis and inhibition of molting in the larva. Ectopic application of a JH analog on *vvl* knockdown larvae delayed the onset of metamorphosis and led to a prolonged larval stage, indicating that Vvl acts upstream of JH signaling. Accordingly, *vvl* knockdown also reduced the expression of a JH biosynthesis gene, *JH acid methyltransferase 3* (*jhamt3*). In addition, ecdysone titer and the expression of the ecdysone response gene, *hormone receptor 3* (*HR3*), were reduced in *vvl* knockdown larvae. The expression of the ecdysone biosynthesis gene *phantom* (*phm*) and *spook* (*spo*) were reduced in *vvl* knockdown larvae in the anterior and posterior halves, respectively, indicating that Vvl might influence ecdysone biosynthesis in both the prothoracic gland and additional endocrine sources. Injection of 20-hydroxyecdysone (20E) into *vvl* knockdown larvae could restore the expression of *HR3* although molting was never restored. These findings suggest that Vvl coordinates both JH and ecdysteroid biosynthesis as well as molting behavior to influence molting and the timing of metamorphosis. Thus, in both vertebrates and insects, POU factors modulate the production of major neuroendocrine regulators during sexual maturation.

## Author Summary

Hormones play major roles in initiating major developmental transitions, such as puberty and metamorphosis. However, how organisms coordinate changes across multiple hormones remains unclear. In this study, we show that silencing the POU domain transcription factor Ventral veins lacking (Vvl)/Drifter in the red flour beetle *Tribolium castaneum* leads to precocious metamorphosis and an inability to molt. We show that Vvl regulates the biosynthesis and signaling of two key insect developmental hormones, juvenile hormone (JH) and ecdysteroids. Vvl therefore appears to act as a potential central regulator of developmental timing by influencing two major hormones. Because POU factors are known as a major regulator of the onset of puberty, POU factors play a major role during sexual maturation in both vertebrates and insects.

## Introduction

Many organisms, including amphibians, echinoderms, marine invertebrates, vertebrates, and insects, undergo dramatic morphological and behavioral changes when they enter metamorphosis or puberty. In holometabolous insects, or insects that undergo complete metamorphosis, the larva molts several times before transforming into a pupa and ultimately into an adult. In mammals, puberty is also associated with morphological changes and reproductive maturation. These dramatic transformations are orchestrated by neuroendocrine changes that occur during postembryonic development of an organism. While the central nervous system (CNS) is known to regulate these endocrine changes [1]–[6], the link between the CNS and the endocrine centers remains poorly understood.

Members of the POU family have been shown to influence the neuroendocrine system during puberty and early development of vertebrates [7]–[13]. POU proteins have a highly conserved POU homeodomain and regulate gene expression by binding to high-affinity octamer sites [8], [14]. Many known POU factors are expressed in cell- or region-specific patterns within the developing CNS, suggesting a role in neural development. In addition, POU factors regulate the onset of puberty in mammals [13], [15].

Because POU domain transcription factors have been found in both vertebrates and invertebrates, their neuroendocrine functions may be conserved across species. Here, we investigated a homolog of the POU domain transcription factor, Ventral veins lacking (Vvl)/Drifter, in the holometabolous insect, *Tribolium castaneum*. Vvl has been shown to regulate the development of the tritocerebrum, CNS, peripheral nervous system and trachea in *Drosophila* embryos [16]–[22]. In addition, Vvl has been shown to regulate the expression of Diapause hormone-pheromone biosynthesis-activating neuropeptide (DH-PBAN) coding gene in the silkworm, *Bombyx mori*, and in the cotton bollworm, *Helicoverpa armigera* [23], [24]. More recently, it has been shown that in *Drosophila*, the endocrine glands, which synthesize metamorphic hormones, and trachea, share the same developmental origin, and that the progenitor cells that give rise to these structures express Vvl [25]. These studies suggest that Vvl, like vertebrate POU factors, may act within the neuroendocrine organs to regulate the biosynthesis of metamorphic hormones.

Many insects undergo three distinct phases of development: the larval, pupal and adult stages. The larval stage is primarily a feeding stage marked by several larval-larval molts (shedding of the cuticle) that enables the larva to grow to a sufficient size. Ecdysteroids and JH are two major developmental hormones involved in the transition from a larva to a pupa [3]. Ecdysteroidogenesis occurs in the prothoracic gland and involves the conversion of cholesterol into precursors of 20-hydroxyecdysone (20E), the primary ecdysteroid involved in molting. These precursors are ecdysone (E) in *Drosophila* and beetles, or 3-dehydroecdysone in *Manduca sexta* and a number of other lepidopteran species [26]–[29]. The secretion/synthesis of ecdysone is in turn triggered by prothoracicotropic hormone (PTTH), a neuropeptide released from the corpora cardiaca [3], [30], and by insulin signaling [31], [32]. Inside the prothoracic gland, these signaling pathways regulate the expression of several Halloween family genes, such as *spook* (*spo*), *phantom* (*phm*), *disembodied* (*dib*) and *shadow* (*sad*), which code for cytochrome P450 enzymes necessary for catalyzing a series of reactions that ultimately convert cholesterol into E [26], [33]–[35]. Mutations in these genes lead to lowered ecdysteroid titers in *Drosophila* [33]. The expressions of these genes are regulated dynamically by both PTTH and insulin signaling [34] and correlate with the ecdysteroid titer. Once released into the hemolymph, E is converted to 20E in the peripheral tissues by *shade* (*shd*) [36].

At the target tissues, ecdysteroids act by binding to the nuclear hormone receptor Ecdysone receptor (EcR), a heterodimer with the RXR homolog Ultraspiracle (Usp). Knockdown of EcR or Usp expression leads to disrupted molting and metamorphosis [37]–[40]. Once 20E binds to EcR, a series of ecdysone response genes are activated. Among the first genes to be activated are so-called primary response genes, which include *E74* and *E75* [41]–[44]. Subsequently, these early response genes activate the expression of delayed early genes, such as *HR3* [45], [46]. Silencing the expression of these ecdysone response genes leads to disrupted molting and if silenced during the final instar, the larvae typically arrest their development during the prepupal period, indicating that metamorphosis is incomplete [47]–[49]. These studies show that ecdysteroid signaling is essential for molting and the completion of metamorphosis.

The nature of this ecdysteroid-induced molting depends on JH, a sesquiterpenoid hormone that is secreted from the corpora allata [50]. JH is known as a “*status quo*” hormone because it prevents progression to the next life stage after a molt [51]. In holometabolous insects, JH is present at high levels during the larval stages (instars) and prevents progression to the pupal stage during a molt. JH titers are known to be regulated by a complex interplay between JH synthesis, degradation and sequestration by JH binding proteins [52]. Only when JH levels drop in the last instar can ecdysone induce metamorphosis. That JH decline is essential for the initiation of metamorphosis has been illustrated through several distinct approaches. First, in many insects, application of JH during the penultimate instar induces a supernumerary molt [53]. [54]–[56]. Second, previous studies have shown that the removal of the JH-producing corpora allata can induce precocious metamorphosis with larvae exhibiting pupal characteristics before entering the final larval instar [57]. In addition, recent studies have demonstrated that the basic helix-loop-helix (bHLH)- Per-Arnt-Sim (PAS) domain protein encoded by *Methoprene-tolerant* (*Met*) plays a key role in mediating sensitivity to JH and likely acts as a JH receptor in *Tribolium* [58]–[60]. When *Met* is knocked down in *Tribolium*, larvae undergo precocious metamorphosis, a few larval molts earlier than normal [59], [61], [62], and develop into miniature adults. Finally, silencing of the JH biosynthesis gene, *JH acid methyltransferase 3* (*jhamt3*), in *Tribolium* leads to precocious metamorphosis, again after fewer larval molts than normal, resulting in the formation of miniature adults [63]. The expression of this gene tracks the JH titer closely [63], and JHAMT is thought to act on the rate-limiting step for the series of biochemical reactions that ultimately results in the formation of active JH [64]. The regulation of JH biosynthesis is clearly an integral part of the regulation of timing of metamorphosis. However, how the expression of the key JH biosynthetic enzymes is regulated remains poorly understood. To summarize, the timing of metamorphosis is regulated by the dynamic titers of ecdysteroids and JH. Ecdysteroids are required for molting and their removal leads to disrupted molting and failure to initiate or complete metamorphosis. In contrast, JH is required to maintain the larval stage and its removal leads to precocious metamorphosis because in its absence, ecdysone induces metamorphosis even if the larva has undergone fewer larval-larval molts than in the wildtype.

In this study, we investigated the role of the POU domain transcription factor Vvl in coordinating the metamorphic hormones in *Tribolium*. *Tribolium* is well suited for the study of metamorphic regulation because of its sequenced genome and amenability to RNA interference (RNAi). Moreover, JH in *Tribolium* plays a prominent role in determining the number of molts prior to metamorphosis, facilitating the study of the role of developmental hormones in the regulation of metamorphic timing [59], [61], [63]. In contrast, *Drosophila* has a fixed number of instars, and topical application of JH does not lead to additional larval molts [65].

To determine the function of Vvl in *Tribolium*, we knocked down the expression of *vvl* and found that this resulted in precocious metamorphosis. These animals also had lowered expression of the JH response gene, *krüppel-homolog 1* (*kr-h1*), which could be restored with topical application of JH. Knockdown of *vvl* also resulted in the reduced expression of *jhamt3*, a key regulator of JH biosynthesis. In addition, *vvl* knockdown led to an inability to molt and a corresponding reduction in ecdysone levels, and the expressions of ecdysone biosynthesis genes and the ecdysone-response gene, *HR3*. The expression of *HR3* but not the molting defects could be rescued by injection of 20E. Our results suggest that Vvl may act as a nexus between JH and ecdysone biosynthesis.

## Results

### Vvl ortholog in *Tribolium*

**Figure 1:**
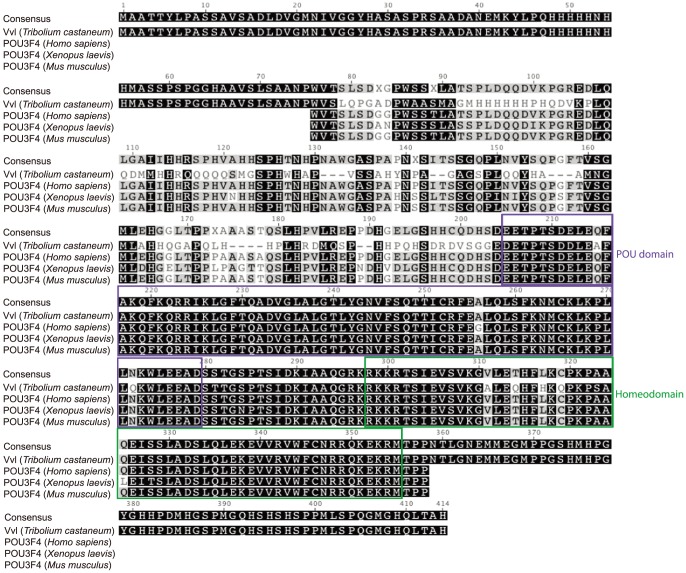
Sequence alignment of the conserved regions of *Tribolium* Vvl and the vertebrate POU3F4 proteins. Sequences were downloaded from NCBI and aligned using the Geneious 6 software. The POU domains and homeodomains are highlighted.

We have identified one single ortholog of Vvl in the *Tribolium* Genome Base (http://www.beetlebase.org). Phylogenetic analysis of *Tribolium* POU factors confirms that the *Tribolium* Vvl clusters with both *Drosophila* and *Bombyx* Vvl homologs (Figure S1). To compare the Vvl ortholog from *Tribolium* to those of other invertebrates and vertebrates, the *Tribolium* Vvl protein sequence was identified in the *Tribolium* Genome Base and blasted in Geneious (http://www.geneious.com/). The POU region of the *Tribolium* Vvl consists of a POU domain and a homeodomain. The amino acid sequence of this region is highly conserved with those found in other species, such as Vvl in *Drosophila melanogaster* (98% sequence identity), POU-M2 in *Bombyx mori* (98% sequence identity), POU3F4 in *Mus musculus* (93% sequence identity), *Xenopus laevis* (93% sequence identity) and *Homo sapiens* (93% sequence identity) (Figure 1; Figure S2).

### Expression of *vvl* in wildtype *Tribolium*

**Figure 2:**
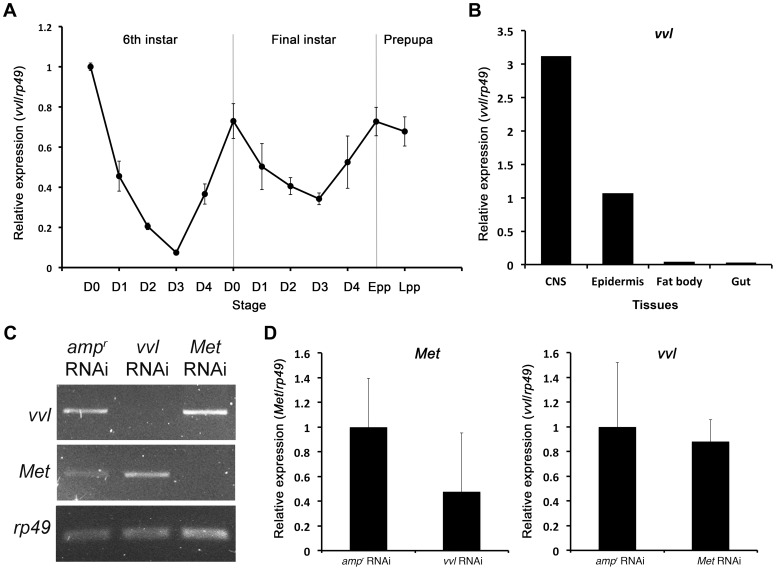
Expression profile of *vvl* and knockdown verification for RNA interference. (A) Expression profile of *vvl* during the late larval and prepupal stages of *Tribolium.* Expression profile was determined by qPCR of *vvl* from whole body sixth and seventh instars and prepupae. *Ribosomal protein 49 (rp49)* was used as the internal control. For all of the treatments, each sample consisted of RNA pooled from five sixth instars, three seventh instars and three prepupae. Three biological replicates were used per treatment, and each sample was run in triplicates. (B) Expression of *vvl* in the CNS, epidermis, fat body and gut of day 0 seventh instar larvae. mRNA was isolated from tissues pooled from 20 individuals. (C) Knockdown verification of *vvl* and *Met* in early prepupae. Expression profiles for *vvl*, *Met*, and *rp49* (control) in *amp^r^*, *vvl*, and *Met* dsRNA-injected animals. Cycle numbers for *vvl*, *Met* and *rp49* were 34, 35, and 28, respectively. (D) Quantitative real-time PCR data showing the expression of *Met* (left) and *vvl* (right) in *vvl* and *Met* knockdown prepupae, respectively.

To determine the expression profile of *vvl* in *Tribolium*, qPCR was used to amplify *vvl* in cDNA obtained from the whole body mRNA extracts of the sixth and final instar larvae, and prepupae (Figure 2A). The expression of *vvl* was the highest on day 0 of the sixth instar and then dropped to a low level by day 3. The expression then increased to a high level at the time of the molt to the final instar. During the final instar, the *vvl* expression decreased gradually until the larva entered the prepupal stage when *vvl* expression increased again to a high level. The fluctuations observed are similar to the fluctuations of ecdysteroids and JH observed during the penultimate and final instars in other insects [51]. Thus, *vvl* expression might be correlated with endocrine signaling.

To further investigate tissue specific expression of *vvl*, *vvl* expression was analyzed in the CNS/corpora allata complex, epidermis, fat body and gut of day 0 seventh instar larvae using qPCR. The expression of *vvl* was the highest in the CNS/corpora allata complex (Figure 2B). Vvl was also expressed in the epidermis. Very low amounts of *vvl* mRNA were detected in the fat body and the gut. If *vvl* influences endocrine signaling, then one might expect that its removal would disrupt molting and/or the metamorphic transition.

### *vvl* knockdown causes precocious metamorphosis

**Figure 3:**
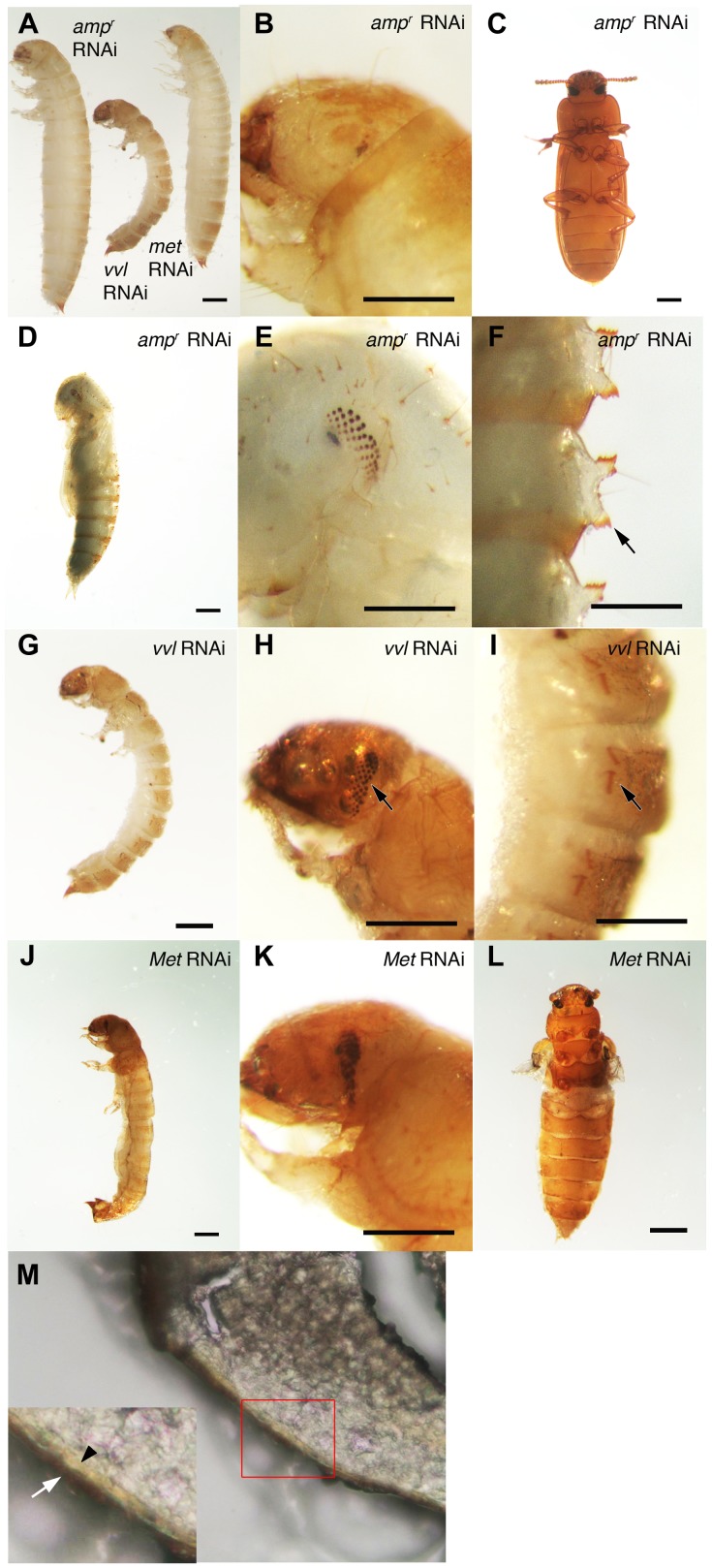
Phenotypic effect of dsRNA injections in fifth instar larvae. (A) Size comparison of *amp^r^*, *vvl* and *Met* dsRNA-injected prepupae. (B) *amp^r^* dsRNA-injected prepupal eyes. (C) *amp^r^* dsRNA injected normal adult. (D) *amp^r^* dsRNA-injected pupa. (E) *amp^r^* dsRNA-injected pupal eyes. (F) *amp^r^* dsRNA-injected pupal gin traps (arrow). (G) *vvl* dsRNA-injected prepupa. (H) *vvl* dsRNA-injected prepupa with compound eyes (arrow) developing underneath the cuticle. (I) *vvl* dsRNA-injected prepupa with pupal gin traps (arrow) developing underneath the cuticle. (J) *Met* dsRNA-injected prepupa. (K) *Met* dsRNA-injected prepupal eyes. (L) *Met* dsRNA-injected eclosed adult. (M) Cross section of a *vvl* dsRNA-injected prepupa that failed to pupate. (Inset) The old darker larval cuticle (white arrow) is attached to the newly synthesized pupal cuticle (black arrowhead). Scale bars indicate 0.5 mm.

**Figure 4:**
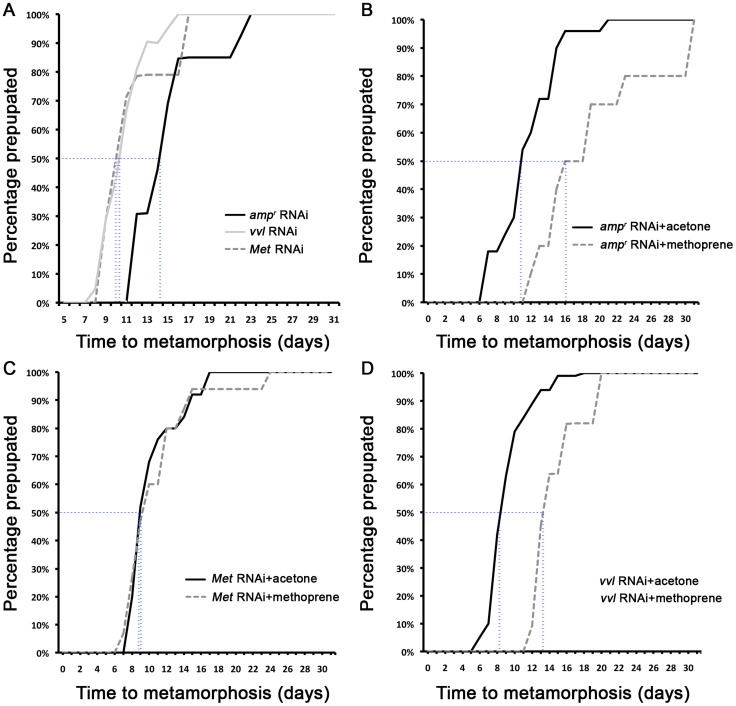
The timing of metamorphosis initiation in larvae treated with *vvl* dsRNA and 5 ng methoprene. (A) Timing of prepupa formation in dsRNA injected fifth instars. (B–D) Timing of prepupa formation in dsRNA-injected fifth instars treated with acetone and methoprene. *amp^r^* RNAi (B), *Met* RNAi (C) and *vvl* RNAi (D) indicate animals injected with *amp^r^* dsRNA, *Met* dsRNA and *vvl* dsRNA, respectively. The time to the onset of prepupal period was recorded. All animals were maintained at 29°C and 50% humidity.

To investigate the functions of Vvl during *Tribolium* development, *vvl* double-stranded RNA (dsRNA) was injected into day 0 fifth instar *Tribolium* larvae. All animals injected with *vvl* dsRNA initiated precocious metamorphosis and entered the prepupal stage without molting (n = 15; Figures 3 and 4A; Table 1). In contrast, control larvae injected with *ampicillin-resistance* (*amp^r^*) dsRNA at the beginning of the fifth instar molted at least two more times before initiating metamorphosis, typically at the end of the seventh instar stage (n = 16). After larvae were injected with *amp^r^* dsRNA, the larvae took approximately 12 days to enter the quiescent stage; in contrast, when animals were injected with 0.5 µg of *vvl* dsRNA, the timing of metamorphosis was shifted about four days earlier (Figure 4A). Because the animals injected with *vvl* did not molt to subsequent larval instars but rather initiated metamorphosis without molting (Table 1), the *vvl* dsRNA-injected prepupae were much smaller than the *amp^r^* dsRNA-injected prepupae (Figure 3A). The larvae injected with *vvl* dsRNA arrested at the prepupal stage, but the pupal characters, such as compound eyes and gin traps, eventually developed under the larval cuticle (Figures 3B–I). When these animals were sectioned, the old larval cuticle and the newly synthesized pupal cuticle appeared to be attached to each other, indicating that these animals fail to complete apolysis (Figure 3M). The *vvl* dsRNA-injected animals never developed into adults. To determine whether this effect was indeed due to the specific effect of *vvl* knockdown, dsRNA targeted to another region of the *vvl* gene was injected into fifth instar larvae. Similar precocious metamorphosis was observed (Figure S3), indicating that the precocious metamorphosis observed in this study is due to knockdown of *vvl* and not another gene.

**Table 1:** Phenotypic effects of dsRNA injection into fifth instar larvae.

dsRNA injected	N	Metamorphosis after…				Stage attained					
		0 molt	1 molt	2 molts	3 molts	Larva	Prepupa			Pupa	Normal Adult
							No complex eye development	Compound eye development	Adult cuticle formation		
*amp^r^*	16	0	2	11	3	3	0	1	0	1	11
*vvl*	15	15	0	0	0	1	3	11	0	0	0
*Met*	21	3	18	0	0	0	0	5	14*	2	0

*includes animals that eclosed as abnormal adults without pupating.

The precocious metamorphosis seen in *vvl* dsRNA-injected larvae suggested the possibility that JH signaling might be affected. To determine how *vvl* dsRNA-injected larvae compare with larvae that have reduced expression of the JH receptor Met, *Met* dsRNA was injected into day 0 fifth instar larvae. *Met* dsRNA-injected larvae molted precociously, and a comparison of the mean time to metamorphosis showed that the mean time to metamorphosis in *vvl* and *Met* dsRNA-injected larvae was not significantly different from each other whereas they metamorphosed significantly earlier compared to *amp^r^* dsRNA-injected animals (p<0.005, ANOVA with Tukey HSD). However, in contrast to the *vvl* dsRNA injected animals, most larvae injected with *Met* dsRNA successfully completed a single molt before precociously entering metamorphosis (Table 1). Furthermore, the *Met* dsRNA-injected animals did not arrest their development at the prepupal stage (Figures 3J–L). Nearly 67% of *Met* dsRNA-injected animals (n = 21) began to develop adult tissues in the head and thoracic regions under the old larval cuticle and eventually eclosed as an abnormal adult, similar to the phenotypes reported by Parthasarathy et al (2008) ([61]; Table 1; Figures 3J–L). These observations suggest that *vvl* not only influences JH signaling but might also regulate the molting process.

Semi-quantitative RT-PCR was used to verify knockdown of *vvl* expression in early prepupae after last instar larvae were injected with 0.5 µg of dsRNA. In addition, semi-quantitative RT-PCR and qPCR were used to determine whether knockdown of either *vvl* or *Met* leads to changes in *Met* or expression, respectively. *vvl* mRNA expression level was lower in animals injected with *vvl* dsRNA than in the control larvae injected with *amp^r^* dsRNA and larvae injected with *Met* dsRNA (Figures 2C and 2D). In addition, *Met* expression level was lower in animals injected with *Met* dsRNA than in those injected with *amp^r^* dsRNA or *vvl* dsRNA (Figure 2C and 2D). These results demonstrate that the *vvl* and *Met* expression was effectively reduced and that Met and Vvl do not regulate the mRNA expression of each other.

### *vvl* knockdown leads to a reduction of *jhamt3* expression

The precocious metamorphosis suggested that JH biosynthesis might be impaired in *vvl* knockdown animals. A key JH biosynthesis enzyme is encoded by JHAMT, an enzyme that converts JH acid into JH. This step has been proposed to act as the rate-limiting step of JH biosynthesis [64], and its knockdown in *Tribolium* results in precocious metamorphosis [63]. The *vvl* expression profile described above follows closely the published expression profile of *jhamt3* [63]. Thus, we examined whether the removal of Vvl affects the expression of *jhamt3*.

**Figure 5:**
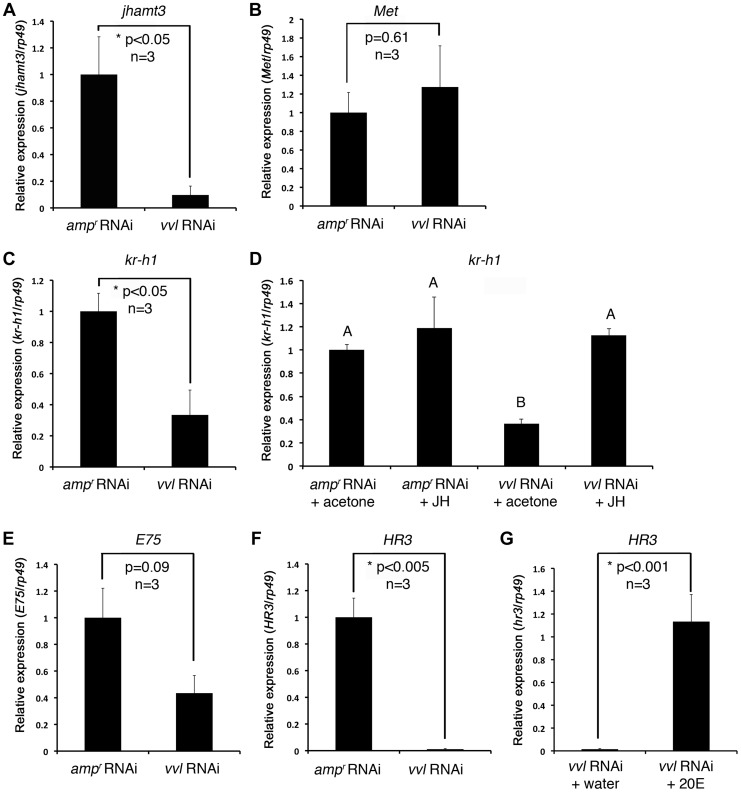
Effect of *vvl* knockdown on genes involved in JH and ecdysone signaling. (A–C) Effect of knockdown of *vvl* on the expression of *jhamt3* (A), *Met* (B) and the JH response gene, *kr-h1* (C), in fifth instar larvae. Three biological replicates were used per treatment, and each sample was run in triplicates. (D) Effect of methoprene application on the expression of *kr-h1* in *vvl* knockdown larvae. *vvl* dsRNA-injected larvae were treated with either acetone (control) or methoprene (15 µg) on day 0 of the fifth instar stage. *amp^r^* dsRNA-injected animals treated similarly were used as a comparison. Four biological replicates were used per treatment, and each sample was run in triplicates. Means not sharing the same letter are significantly different (p<0.05, ANOVA with Tukey HSD test). (E, F) Effect of knockdown of *vvl* on the expression of *E75* (E) and *HR3* (F). (G) Effect of 20E injection on the expression of *HR3* in *vvl* knockdown larvae. Three biological replicates were used per treatment, and each sample was run in triplicates. For (A)–(F), each sample consisted of RNA pooled from five larvae. For (G), each biological replicate consisted of RNA pooled from three larvae. Data are represented as mean +/− SEM.

To determine whether Vvl plays a role in JH biosynthesis, qPCR was performed on day 4 fifth instar *Tribolium* larvae that were injected with either *vvl* or *amp^r^* dsRNA on day 0. The expression of *jhamt3* was significantly decreased when the larvae were injected with *vvl* dsRNA (Figure 5A). In contrast, the expression of *Met* did not differ between *vvl* and *amp^r^* dsRNA-injected larvae (Figure 5B). Thus, *vvl* appears to play a crucial role in JH biosynthesis by influencing the expression of *jhamt3*.

### Precocious metamorphosis of *vvl* dsRNA-injected larvae is rescued by methoprene

If JH biosynthesis, and not JH sensitivity, is affected by *vvl* knockdown, topical application of methoprene should restore the normal timing of metamorphosis. We therefore ectopically applied the JH analog, methoprene, to day 0 fifth instar larvae injected with *vvl* dsRNA. Acetone treatments were used as controls, and *amp^r^* and *Met* dsRNA-injected larvae were also treated similarly for comparison.

**Figure 6:**
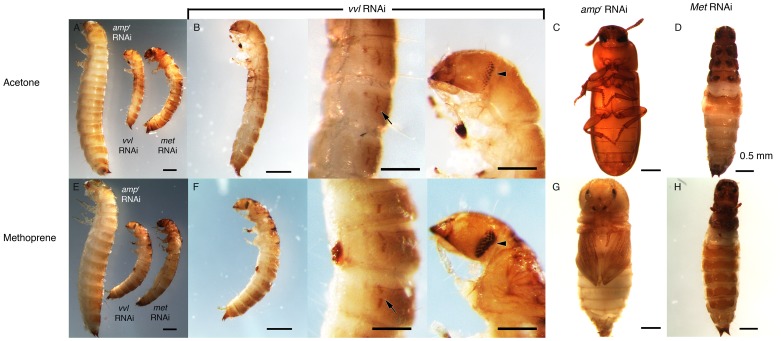
Phenotypic effect of dsRNA injection with methoprene and acetone treatments. (A–D) Acetone-treated animals. (A) Size comparison of *amp^r^*, *vvl* and *Met* dsRNA injected prepupae treated with acetone. (B, left) *vvl* dsRNA-injected prepupa. (B, middle) *vvl* dsRNA-injected prepupa with gin traps (arrow) developing underneath the cuticle. (B, right) *vvl* dsRNA-injected prepupa with complex eyes (arrowhead) developing underneath the cuticle. (C) *amp^r^* dsRNA-injected adult. (D) *Met* dsRNA-injected eclosed adult. (E–H) Methoprene-treated prepupae. (E) Size comparison of *amp^r^*, *vvl* and *Met* dsRNA-injected animals treated with methoprene. (F, left) *vvl* dsRNA-injected prepupa. (F, middle) *vvl* dsRNA-injected prepupa with gin traps (arrow) developing underneath the cuticle. (F, right) *vvl* dsRNA-injected prepupa with complex eyes (arrowhead) developing underneath the cuticle. (G) *amp^r^* dsRNA-injected arrested pupa. (H) *Met* dsRNA-injected eclosed adult. Scale bars represent 0.5 mm.

All controls injected with *amp^r^* dsRNA and treated with acetone molted into pupae and formed normal adults (Table 2; Figures 6A and 6C). Application of 5 ng methoprene to day 0 fifth instar larvae injected with *amp^r^* dsRNA caused supernumerary molts (extra larval molts after the eighth instar) in seven out of 14 larvae (Table 2). Five of the methoprene-treated *amp^r^* dsRNA-injected animals failed to progress beyond the larval stage, but nine were able to undergo metamorphosis (Figure 6E); however, most larvae that underwent metamorphosis arrested their development and died as either prepupae or pupae (Table 2; Figure 5G). The time to metamorphosis was significantly delayed relative to those treated with acetone (Figure 4B; p<0.005, Student's t-test). When 15 µg of methoprene was applied to day 0 fifth instar larvae, the majority of the larvae stayed in the larval stage and did not pupate (n = 13/14).

**Table 2:** Effects of ectopic application of methoprene on developmental timing in larvae injected with dsRNA as fifth instars.

dsRNA injected	N	Acetone or methoprene	Metamorphosis after….					Stage attained					
			0 molt	1 molt	2 molts	3 molts	4 molts	Larva	Prepupa			Pupa	Adult
									No compound eye development	With compound eye development	Adult cuticle formation		
*amp^r^*	19	Acetone	0	6	11	2	0	3	0	2	0	1	13
*amp^r^*	14	5 ng methoprene	0	2	5	5	2	5	0	2	0	7	0
*vvl*	20	Acetone	20	0	0	0	0	1	0	19	0	0	0
*vvl*	16	5 ng methoprene	16	0	0	0	0	6	3	7	0	0	0
*Met*	25	Acetone	10	15	0	0	0	0	1	8	11	4	1
*Met*	16	5 ng methoprene	11	5	0	0	0	1	1	4	8	2	0

In contrast, day 0 fifth instar larvae injected with *vvl* dsRNA and treated with 5 ng methoprene were still unable to molt (n = 16), and most died as prepupae (n = 10; Table 2). These prepupae ultimately developed pupal-like characteristics underneath the larval cuticle (Figures 6B and 6F). Ectopic application of methoprene delayed the timing of metamorphosis relative to those treated with acetone (Figure 4D; p<0.0001, Student's t-test). When the concentration of methoprene was increased to 15 µg, none of the larvae initiated metamorphosis and eventually died without ever molting (n = 9). The larvae survived on average for 23.9 days without molting.

As a comparison, day 0 fifth instar larvae were also injected with *Met* dsRNA and treated with acetone or 5 ng methoprene. For both treatments, the majority of *Met* dsRNA-injected animals underwent metamorphosis and developed into prepupae or eclosed as adults (Table 2; Figures 6A, 6D, 6E and 6H). In agreement with previous studies suggesting that Met is a receptor for JH, there was no significant difference between the timing of metamorphosis in larvae treated with acetone or 5 ng methoprene (Figure 4C; p = 0.61, Student's t-test), indicating that *Met* knockdown animals are insensitive to JH. Similarly, all *Met* knockdown animals treated with 15 µg of methoprene metamorphosed precociously, unlike those injected *amp^r^* or *vvl* dsRNA and treated with 15 µg of methoprene (n = 13/14). Taken together, the results support the notion that Vvl acts on JH biosynthesis but not JH reception to regulate the timing of metamorphosis.

### Expression of *kr-h1* is reduced in *vvl* dsRNA-injected larvae

To determine whether Vvl influences the expression of a downstream target gene of the JH pathway, qPCR analysis was performed on day 4 fifth instar *Tribolium* larvae that were previously injected with *vvl* dsRNA and treated with either acetone (control) or 15 µg methoprene on day 0 of the fifth instar. *amp^r^* dsRNA-injected animals treated similarly were used as a comparison. *kr-h1* expression was reduced in the *vvl* dsRNA-injected larvae in comparison to control *amp^r^* dsRNA-injected larvae (Figure 5C). In larvae that were ectopically treated with 15 µg methoprene following injection of *vvl* dsRNA on day 0 of the fifth instar, the expression level of *kr-h1* was restored to a level similar to that of the *amp^r^* dsRNA-injected animals treated with methoprene (Figure 5D). Thus, knockdown of *vvl* results in the downregulation of *kr-h1* expression which can be rescued with the ectopic application of methoprene. These results further support the notion that JH biosynthesis rather than reception is influenced by Vvl.

### *vvl* knockdown larvae have reduced expression of ecdysone response genes

The inability of *vvl* dsRNA-injected larvae to molt indicated a potential disruption of ecdysteroid signaling pathway, which is required for molting. To determine whether Vvl is required for the activation of ecdysone response genes that are associated with molting, we examined the expression of two such genes, *E75* and *HR3* [48], in *vvl* dsRNA- and *amp^r^* dsRNA-injected larvae four days after injection, just prior to their molt to the sixth instar in control larvae. The expression level of the ecdysone inducible early gene, *E75*, was not significantly reduced in *vvl* dsRNA-injected larvae (Figure 5E). However, the ecdysone-inducible gene, *HR3*, was dramatically reduced in *vvl* dsRNA-injected larvae (Figure 5F). We do not know why E75 did not decrease significantly in *vvl* knockdown animals. One reason might be that *HR3*, further downstream in the transcriptional cascade, is more sensitive than E75 to sustained Vvl-mediated changes in ecdysteroid secretion or action. Nevertheless, our results taken together suggest that ecdysteroid signaling is disrupted in *vvl* knockdown larvae. Thus, Vvl may have a dual role as both an activator of JH signaling and as a regulator of molting through its influence on ecdysteroid signaling.

### Vvl knockdown larvae have lowered ecdysteroid titer

**Figure 7:**
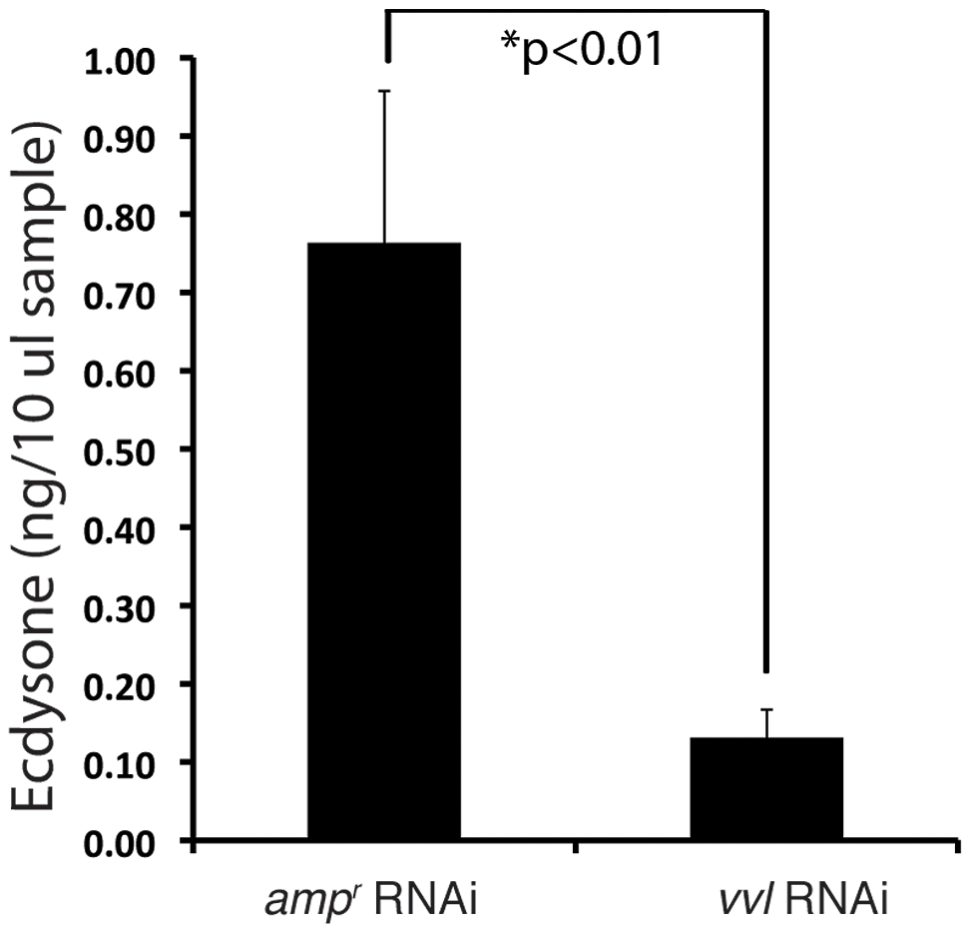
Vvl removal leads to decreased ecdysteroid titer. Ecdysone titers in *amp^r^* and *vvl* dsRNA-injected fifth instar larvae on day 4. A 10 µl sample represents two larval equivalents of extracted ecdysteroids.

To determine whether ecdysteroid titer was affected in response to *vvl* knockdown, ecdysteroid titer was observed on day 4 of the fifth instar after larvae were injected with either *vvl* dsRNA or *amp^r^* dsRNA on day 0 of the fifth instar. We observed that larvae injected with *vvl* dsRNA have a lower ecdysteroid titer relative to *amp^r^* dsRNA-injected larvae (Figure 7; p<0.01, Student's t-test). Together with the lack of molting phenotype and the lowered expression of ecdysone response genes, the lowered ecdysone titer indicates that Vvl influences ecdysteroid biosynthesis.

**Figure 8:**
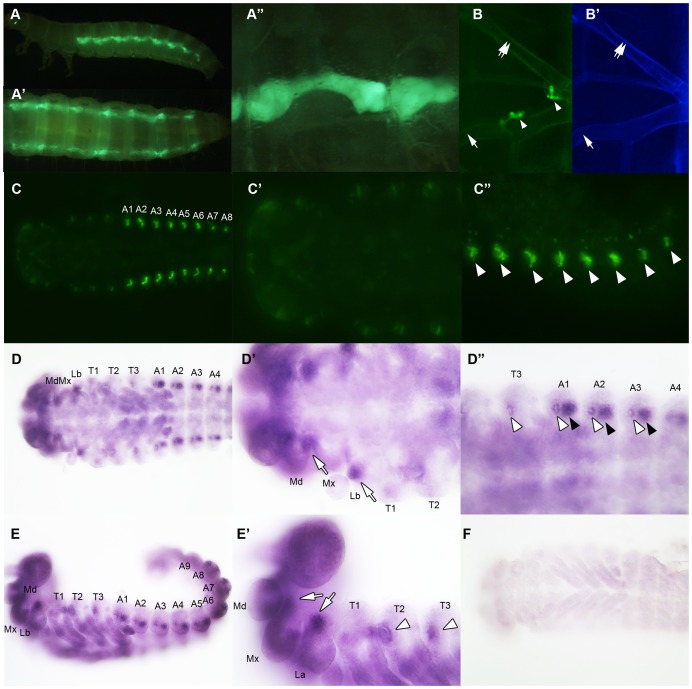
*vvl* expression in embryos and sixth instar larvae. (A–A″) Vvl-GFP expression in the oenocytes of the sixth instar KT817 enhancer trap line. GFP expression in the oenocytes of this strain is driven by a *vvl* enhancer [66]. Lateral (A), dorsal (A′) and close-up (A″) views are shown. (B) Vvl-GFP expression in the dorsal and ventral tracheal trunks in the prothorax of a sixth instar KT817 larva. Double arrow indicates the dorsal tracheal trunk, and single arrow points to the ventral tracheal trunk. Arrowheads point to GFP-positive cells on the trachea. (B′) Autofluorescence of the same structures shown in (B). (C–C″) GFP expression in the embryo of a KT817 enhancer trap line. Ventral view (C), close-up of the anterior end (C′) and close-up of the posterior end (C″) are shown. Arrowheads indicate the oenocytes. (D–D″, E, E′) Ventral (D–D″) and lateral views (E, E′) of *vvl* mRNA expression in the day 1 embryo as detected by *in situ* hybridization. (D′, D″, E′) Close-up of the embryos shown in D and E. White arrows indicate *vvl* expression detected in the maxillary and labial segments. White arrowheads indicate tracheal pits. Black arrowheads indicate the oenocytes. (F) Embryos probed with a control *vvl* sense probe.

A recent study by Burns et al (2012) demonstrated that a GFP enhancer trap line that expresses GFP under the control of the *vvl* enhancer (strain KT817) expresses GFP in the oenocytes during the embryonic and early larval stages [66]. We examined the late sixth instar larvae and found GFP expression in the abdominal structures that most likely correspond to oenocytes (Figures 8A–8A″). Since oenocytes have been shown to be capable of synthesizing ecdysone from cholesterol in another tenebrionid beetle, *Tenebrio molitor* [67], [68], we wondered if the reduction of ecdysone titer might result from the reduced expression of *vvl* in the oenocytes.

**Figure 9:**
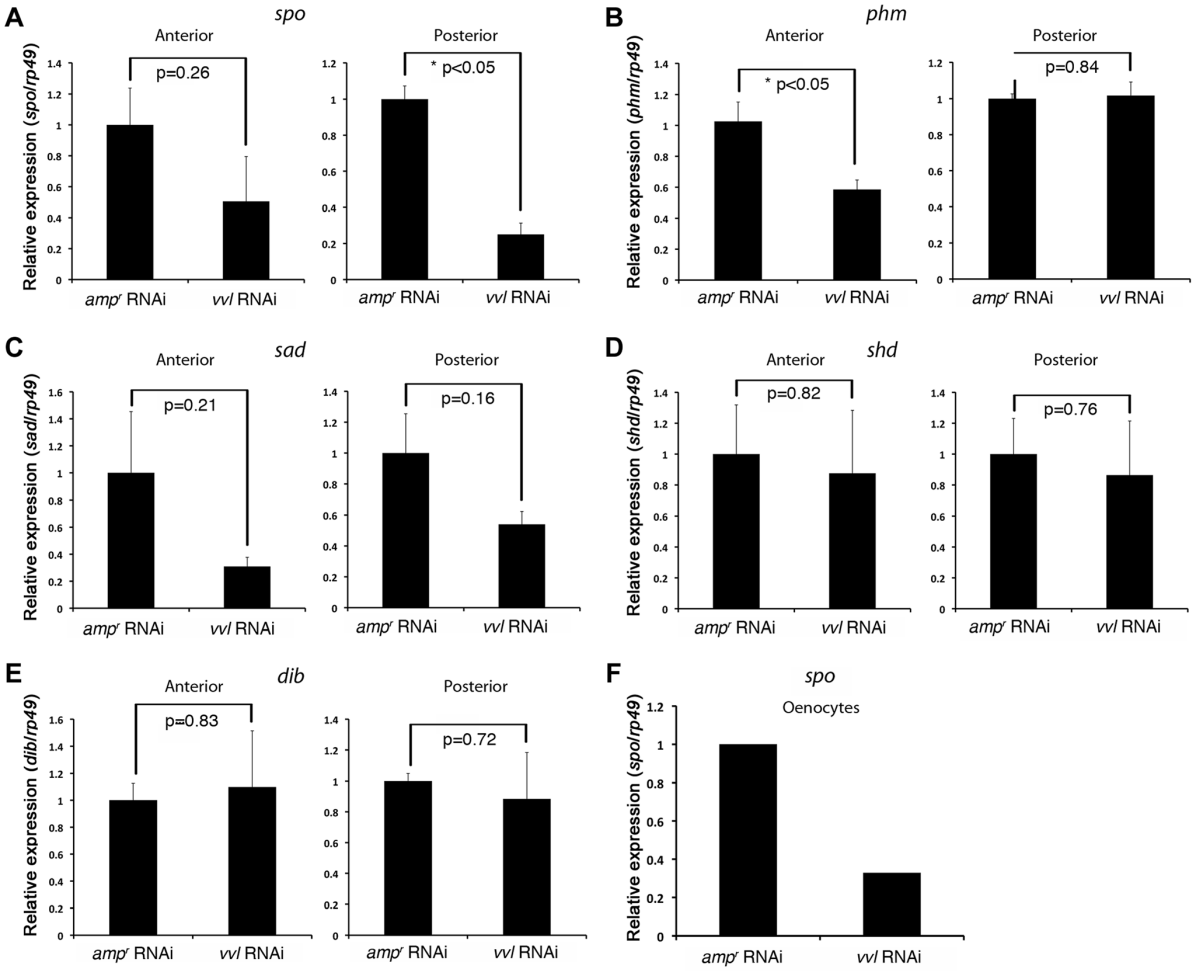
*vvl* knockdown leads to decreased expression of ecdysone biosynthesis genes. (A–E) Ecdysone response gene expression in *amp^r^* and *vvl* dsRNA-injected fifth instar larvae on day 4. Anterior represents expression in the head and thorax. Posterior represents expression in the abdomen. Three biological replicates were used per treatment, and each sample was run in triplicates. Each biological sample consisted of pooled RNA from five larvae. Error bards represent standard error. (F) Relative expression of *spo* expression in the oenocytes of day 4 *amp^r^* and *vvl* dsRNA-injected fifth instar KT817 larvae. Oenocytes from 15 larvae were pooled per treatment.

To determine whether ecdysone biosynthesis was altered in the prothoracic gland or the oenocytes, larvae injected with *vvl* or *amp^r^* dsRNA were bisected and assayed for ecdysone biosynthesis gene expression. We found that the expression of *spo* was significantly reduced in the posterior half of the *vvl* knockdown larvae (Figure 9A). The expression of *spo* in the anterior portion of *vvl* knockdown larvae was not significantly different from that of *amp^r^* knockdown larvae (Figure 9A). To see if expression of *spo* was lowered in the oenoctyes of *vvl* knockdown larvae, day 0 fifth instar KT817 larvae were injected with *vvl* or *amp^r^* dsRNA and oenocytes along with the associated tissues were collected on day 4. We found that the expression of *spo* was lowered in the oenocytes of *vvl* knockdown larvae relative to the *amp^r^* knockdown larvae (Figure 9F). In contrast, we found that *phm* expression was reduced in the anterior portion of *vvl* dsRNA-injected animals relative to those injected with *amp^r^* dsRNA (Figure 9B). The posterior expression of *phm* remained unchanged. The expression of *sad*, *shd* and *dib* did not differ between the *vvl* and *amp^r^* dsRNA-injected animals in both the anterior and posterior portions of the body (Figures 9C–9E). These results suggest that Vvl regulates ecdysone biosynthesis in different glands by influencing the expression of different ecdysone biosynthesis genes.

We also looked for expression of GFP in the anterior portion of the larvae. In Coleoptera, prothoracic glands have been found to be associated with the dorsal tracheal trunk and the ventral tracheal trunk [69]. No obvious expression of GFP was observed in the cells lining the dorsal and ventral tracheal trunks except a few cells, which may correspond to Inka cells (Figures 8B and 8B′). The absence of GFP expression in the PG cells may be due to the KT817 enhancer trap not reflecting the entire repertoire of tissues where the *vvl* gene is active. To determine whether GFP is expressed in all the cells that express *vvl*, *in situ* hybridization was performed on day 1 embryos. In the anterior region, *vvl* mRNA was detected at the base of the maxillary and labial segments as well as the tracheal pits in the T2 and T3 segments (Figures 8D, 8D′, 8E and 8E′). These locations fail to express GFP in the KT817 strain (Figures 8C and 8C′). Similarly, in the abdomen, *vvl* mRNA was detected in the tracheal pits as well as the oenocytes even though no GFP expression was detected in the tracheal pits of the KT817 strain (Figures 8C, 8C″, 8D and 8D″). No staining was observed when the control sense probe was used (Figure 8F). These results suggest that there are additional enhancers that drive expression of *vvl* besides the one that drives GFP expression in the KT817 strain. The expression of *vvl* at the base of the labial and maxillary segments is noteworthy since in the *Drosophila*, *vvl* expressing cells in these segments give rise to the future endocrine glands [25]. While we attempted to stain for *vvl* in the prothoracic glands during the larval stages, we were unable to obtain convincing staining to due to excess background staining inherent to this stage.

### 20E and ecdysteroid agonist RH-2845 are unable to induce a molt in *vvl* knockdown larvae

To determine whether exogenous ecdysteroid agonist can rescue molting defects in *vvl* knockdown larvae, *vvl* dsRNA-injected larvae were treated with either 20E or RH-2845 two days after injection (Table 3). Injection of 1.5 µg or 0.15 µg of 20E led to death in *vvl* dsRNA-injected larvae (Table 3). When the concentration was reduced to 0.015 µg, a few larvae (n = 2/5) were able to survive but did not molt and developed into prepupae. *amp^r^* dsRNA-injected larvae also had high mortality although even at the highest concentration of 20E (1.5 µg), a few larvae survived to undergo a larval-larval molt. Thus, 20E does not appear to rescue the normal molting phenotype in *vvl* knockdown larvae. However, the high number of dead larvae relative to water injected controls (Table 3) suggests that *vvl* dsRNA injected larvae are able to sense 20E even though they cannot molt.

**Table 3:** Effects of 20E or RH-2485 treatments on larvae injected with dsRNA as fifth instars.

Treatment	N	Number molted	Number prepupated without molting	Number dead without molting/prepupal development
*amp^r^* dsRNA + water	5	5	0	0
*amp^r^* dsRNA + 20E (0.015 µg)	7	4	0	3
*amp^r^* dsRNA + 20E (0.15 µg)	6	2	0	4
*amp^r^* dsRNA + 20E (1.5 µg)	7	3	0	4
*vvl* dsRNA + water	6	0	4	2
*vvl* dsRNA + 20E (0.015 µg)	5	0	2	3
*vvl* dsRNA + 20E (0.15 µg)	6	0	0	6
*vvl* dsRNA + 20E (1.5 µg)	9	0	0	9
*amp^r^* dsRNA + acetone	9	7	0	2
*amp^r^* dsRNA + RH-2485 (0.1 µg)	5	5	0	0
*amp^r^* dsRNA + RH-2485 (1 µg)	8	8	0	0
*vvl* dsRNA + acetone	6	0	5	1
*vvl* dsRNA + RH-2485 (0.1 µg)	8	0	4	4
*vvl* dsRNA + RH-2485 (1 µg)	6	0	3	3

To determine whether 20E treatment can rescue *HR3* expression, day 0 fifth instar larvae were injected with *vvl* dsRNA. Two days later, these larvae were injected with either 0.15 µg 20E or water. We found that larvae injected with 20E had significantly higher levels of *HR3* expression (Figure 5G). Thus, exogenous 20E can rescue *HR3* expression in *vvl* knockdown larvae. Our findings show that *vvl* knockdown leads to lowered ecdysteroid titer, which in turn leads to lowered *HR3* expression. However, the failure of 20E to rescue the molting phenotype indicates that *vvl* knockdown impacts the molting process downstream of EcR/Usp.

Since injection of 20E just two days after dsRNA injection is potentially traumatic for the larvae, we decided to also investigate the effect of topical application of an ecdysteroid mimic. In *Tribolium* adults, 1 µg of RH-2845 upregulates the expression of ecdysone response genes, *EcR* and *E75*, indicating that this compound activates of ecdysteroid signaling [70]. *amp^r^* dsRNA-injected larvae molted after 1.6±0.4 days or 3.5±0.6 days when treated with either 0.1 µg or 1 µg of RH-2845, respectively (Table 3). In contrast, larvae injected with *vvl* dsRNA could not molt even when they were treated with either 0.1 µg or 1 µg of RH-2845 (Table 3). A high rate of mortality was also observed when these larvae were treated with RH-2845 (n = 4/8 and 3/6 for 0.1 µg and 1 µg of RH-2845, respectively). The remaining larvae all became prepupae without molting (n = 4/8 and 3/6 for 0.1 µg and 1 µg of RH-2845, respectively). While the mechanism by which RH-2485 acts as an ecdysteroid agonist remains unknown, the fact that RH-2485 is unable to induce a molt in the absence of Vvl supports the notion that Vvl is in part required for mediating the organism's molting behavior, in addition to its role in regulating ecdysone biosynthesis.

## Discussion

In this study, we determined the effects of knocking down *vvl* in *Tribolium* and elucidated a possible mechanism by which the gene interacts with the physiological regulation of development. Use of dsRNA-mediated expression knockdown revealed that suppression of *vvl* results in precocious metamorphosis. We also propose that Vvl acts upstream of JH signaling and that its expression is required for *jhamt3* expression. In addition, we found that Vvl influences ecdysteroid biosynthesis and signaling to regulate molting.

### Vvl regulates the onset of metamorphosis by regulating JH biosynthesis

**Figure 10:**
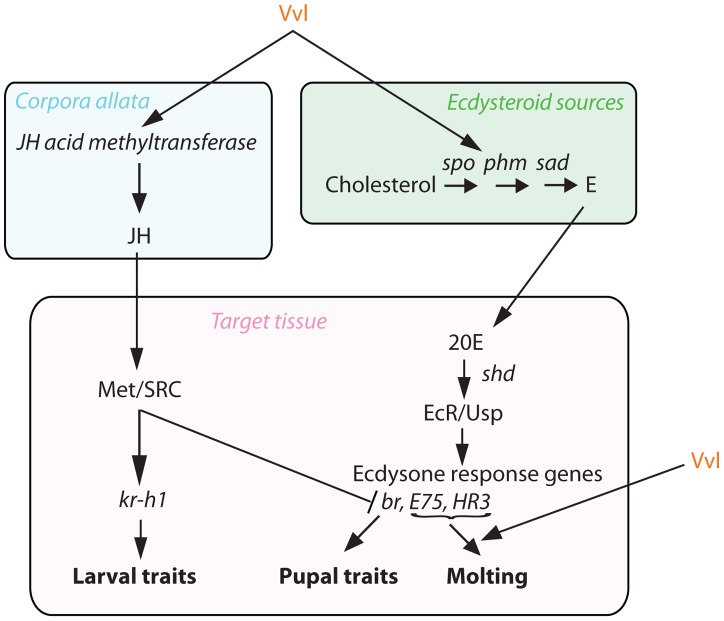
Proposed mechanism of Vvl action. Vvl influences JH production via *jhamt* expression and hence the timing of metamorphosis. Vvl also regulates the biosynthesis of ecdysteroids and the molting pathway.

In the present study, we found that fifth instar animals injected with *vvl* dsRNA underwent premature metamorphosis. The precocious metamorphosis observed in *vvl* dsRNA-injected larvae indicates that these larvae have disrupted JH signaling which results in precocious pupal commitment (Figure 10). Consistent with this interpretation, we found that knockdown of *vvl* expression in the fifth instars causes a down-regulation of the expression of the JH-response gene *kr-h1* [71]. In addition, we found that *vvl* knockdown animals were not resistant to methoprene as seen in *Met* knockdown animals. The delay in metamorphosis observed in these methoprene-treated *vvl* knockdown larvae suggests that *vvl* does not affect the sensitivity to JH. Similarly, ectopic application of methoprene was able to upregulate *kr-h1* expression in *vvl* knockdown animals. Because *kr-h1* is a direct downstream target of Met [58], [71], the down-regulation of *kr-h1* observed in *vvl* knockdown animals and the rescue of its expression with ectopically applied methoprene indicate that *vvl* knockdown causes a decrease in JH biosynthesis by acting upstream of *kr-h1*. Because the expression of *jhamt3*, a JH biosynthesis enzyme, is decreased in *vvl* RNAi animals, Vvl likely influences the rate of JH biosynthesis by influencing the transcription of *jhamt3* directly or by regulating an upstream regulator of *jhamt3* expression. In agreement with this view, *vvl* and *jhamt3* whole body expression profiles are similar (this study; [63]). Our study suggests that this transcription factor plays a key role in regulating the timing of JH biosynthesis. In support of this, we have detected high levels of *vvl* mRNA in day 0 seventh instar CNS/corpora allata complex. While we were unable to distinguish between the expression of *vvl* in the CNS and that in the corpora allata, our finding suggests an intriguing possibility that Vvl might function as a mediator between the nervous system and JH production, potentially linking the two systems to coordinate the onset of metamorphosis.

### Vvl influences ecdysone biosynthesis and its signaling

All larvae injected with *vvl* dsRNA underwent precocious metamorphosis without molting, while *Met* dsRNA-injected animals almost always molted before metamorphosis (Table 1). In addition, although *jhamt3* dsRNA-injected larvae undergo precocious metamorphosis as seen in *vvl* knockdown larvae, *jhamt3* knockdown animals do not show any molting defects and eclose into miniature but externally complete adults [63]. Thus, Vvl suppression might have an additional inhibitory effect on the ecdysteroid signaling pathway, a key regulator of molting. Consistent with this notion, we found that ecdysone titer and the expression of the ecdysone response gene *HR3* were reduced in *vvl* knockdown larvae. This lowered level of *HR3* could be rescued by injection of 20E, further supporting the notion that the *vvl* is required for ecdysteroid biosynthesis. In *Bombyx*, a homolog of Vvl, BmPOUM2, has been shown to be required for metamorphosis. However, when the expression of this gene was knocked down during the wandering stage of *Bombyx*, the expression of ecdysone response genes was not altered [72]. This difference may be due to a difference in the species used or because Vvl removal during wandering is too late to detect an effect on ecdysone biosynthesis or response genes.

We also observed that the expression of the ecdysone biosynthesis genes *phm* and *spo* were lowered in the anterior and posterior halves of the *vvl* knockdown larvae, respectively (Figure 9). The latter observation is consistent with the expression of GFP in the oenocytes of the KT817 GFP enhancer trap line, which expresses GFP under the control of a *vvl* enhancer [66]. In several insect species, including another tenebrionid beetle, *Tenebrio molitor*, oenocytes are capable of producing ecdysone from cholesterol [67]. We have found that when *vvl* is knocked down, *spo* expression decreases. Thus, our observations support the notion that Vvl in the oenocytes regulates ecdysteroid production. However, it is possible that Vvl might target additional ecdysteroidogenic tissues besides oenocytes [68], [73].

We were unable to detect GFP expression in the cells lining the ventral and dorsal tracheal trunk where prothoracic gland cells are typically found [69]. The comparison between embryonic *vvl* expression and embryonic GFP expression in the KT817 line suggests that *vvl* expression in the prothoracic glands and oenocytes are regulated by distinct enhancers. The expression of *vvl* in the labial and maxillary segment of the embryo is similar to what has been observed in *Drosophila*. In *Drosophila*, these *vvl*-expressing cells give rise to the prothoracic glands and the corpora allata. Thus, we suggest that the *vvl* expressing cells in the labial and maxillary segments likely also contribute to the endocrine organs in *Tribolium*.

While we cannot be certain about the mechanism by which ecdysone biosynthesis gene expression is modulated in the different glands, our findings suggest that ecdysone biosynthesis genes are regulated differently in the various endocrine structures. This is not surprising considering that in adults, ecdysone biosynthesis genes are activated differently in the accessory glands and the ovaries [35]. The modular nature of gland specific regulation of hormonal production is probably very common. Future studies should investigate the relative contributions of ecdysteroids from the prothoracic gland and other endocrine sources during larval development.

In addition, the inability of exogenous 20E and RH-2485 to rescue the molting defects despite being able to rescue *HR3* expression suggests that Vvl might affect a component of the ecdysis pathway, such as ecdysis triggering hormone or eclosion hormone [74]–[77]. Our observation of *vvl*-GFP expression in the Inka cell-like cells in the KT817 line is also consistent with this notion since Inka cells have been shown to produce ecdysis triggering hormone in a several insects [77], [78]. The expression of ecdysis triggering hormone is influenced by circulating levels of ecdysteroids and EcR [76], [79], [80]. Preliminary findings show that Vvl binds to EcR/Usp through a GST-pulldown assay (Figure S4). This suggests a possibility that Usp and EcR heterodimerize with Vvl to regulate the expression of ecdysis triggering hormone (Figure S4). However, additional studies, such as ChIP assays, are necessary to definitively determine how Vvl modulates target genes.

### POU factors may regulate metamorphosis in a similar manner as reproductive maturation in vertebrates

Our findings show that Vvl regulates both ecdysone and JH signaling, the two hormonal signaling pathways that regulate metamorphosis (Figure 10). Thus, Vvl may couple the two hormonal pathways to coordinate the timing of metamorphosis in insects. Specifically, we propose that a switch in the expression of Vvl signals the initiation of metamorphosis by regulating the dynamic fluctuations of both of these developmental hormones. Whether Vvl regulates the two hormones separately or relays a change from one to another remains unclear at present.

It has been suggested that puberty in humans and other mammals is another form of metamorphosis. Although seemingly different, both processes are marked by dramatic changes in physiology. Puberty in humans is initiated by endocrine changes in response to increased pulsatile release of gonadotropin releasing hormone (GnRH) from the hypothalamus. In vertebrates, POU factors have been shown to regulate neuropeptide expression. In particular, GnRH expression is regulated by POU factors, which bind to the promoter and enhancer sequences of the GnRH gene. For example, the POU domain transcription factor Oct-6 (POU3F1) represses GnRH activity, delaying the onset of puberty [10]. In the absence of Oct-6, GnRH is expressed, triggering pubertal development.

Our study shows an intriguing similarity between the transcriptional regulation of the key neuroendocrine regulators of insect metamorphosis and mammalian puberty. Most certainly, these two processes have evolved independently and are not homologous. However, given the substantial sequence conservation of POU domain proteins across various metazoan taxa, future studies should investigate whether POU factors play a role in reproductive maturation in other metazoan taxa. Since the corpora allata and the prothoracic gland along with the trachea all express *vvl* during *Drosophila* embryogenesis, it has been suggested that these Vvl-specified structures have a common origin in a proto-arthropod [25], [81]. In light of the endocrine functions we identified in our study, we suggest that *vvl* had an ancient endocrine function, and its expression in these endocrine structures was subsequently retained as corpora allata and prothoracic glands evolved.

## Materials and Methods

### Animal husbandry

Wildtype *Tribolium* strain GA-1 was obtained from Dr. Richard Beeman (USDA ARS Biological Research Unit, Grain Marketing & Production Research Center, Manhattan, Kansas). The KT817 GFP enhancer trap line was obtained from the Kansas State University *Tribolium* Stock Center [82]. All beetles were raised on organic whole-wheat flour fortified with 5% nutritional yeast at 29°C and 50% humidity.

### RNA isolation and cDNA synthesis

*Tribolium* RNA from various larval instars was isolated by homogenizing the tissues in TRIzol (Life Technologies, Carlsbad, CA). The RNA sample was treated with DNAse and converted to complementary DNA (cDNA) using reverse transcriptase (Thermo Scientific, Waltham, MA). The *vvl* gene sequence was obtained from NCBI (NM_001145913). Primers were designed to amplify particular regions of interest in the synthesized cDNA using polymerase chain reaction (PCR) (Table 4).

**Table 4:** Forward (FW) and reverse (RV) primers used for dsRNA synthesis, expression profiling and knockdown verification.

dsRNA synthesis		
*gene*		Primer 5′ → 3′
*vvl* *	FW	GTCTCCTCGGCTCATTACA
	RV	GTCCGCTTGCGTAAATCC
*vvl* (for confirmation)	FW	TACCTTCCGGCGAGCAGT
	RV	CCATGTGGTGGTTGTGATG
*Met* b	FW	GAAGCTTCAAGAGAGGAATATG
	RV	TTTCAACAGTTCCCTGGTCG
**Primers used for knockdown verification and ** ***in situ*** ** hybridization**		
*vvl*	FW	CACCATCACAACCACCACA
	RV	ATTCCCTCCATCATCTCGTT
*Met*	FW	GAGCAGTTGGGTGGTTTTC
	RV	CCGCTTCTTCATTTCGTTT

QRT-PCR primers		
*gene*		Primer 5′ → 3′
*jhamt3* a	FW	CATCTCGCCCTATCACCATTCG
	RV	CCGCTGAAACCGATTTTGACAA
*rp49*	FW	TGACCGTTATGGCAAACTCA
	RV	TAGCATGTGCTTCGTTTTGG
*kr-h1* d	FW	GTTTGCTCCAAGGGGTTCACG
	RV	GGTTGTAGCCGAAGGATTTGCC
*vvl*	FW	GAGCAGCACTTCCACAAACAG
	RV	GGCATTCCCTCCATCATCTC
*Met* c	FW	GGGAAAGCAAAGGATCATCA
	RV	AAGGCCTTCTTGCTCACTCA
*E75* e	FW	CGGTCCTCAATGGAAGAAAA
	RV	TGTGTGGTTTGTAGGCTTCG
*HR3* e	FW	CCGTGCAAAGTATGTGG
	RV	GTCGGCAGTATTGACATC
*phm* f	FW	AAGAATGTGTGTCGGTGATGAA
	RV	TCGTGAGGTTTCGGAGTTAGTG
*spo* f	FW	GGGACGAGCCTGGACTGTT
	RV	CCCGTGCTGAAAGGAATGA
*sad* f	FW	GCTAAGAGCCCGCAAATCC
	RV	GGTAAAGCCGCAAAGTCTCCT
*shd* f	FW	GGTCAACGAACAAGGTGAGG
	RV	GAGTCGGTCTGCGATGTAGTTT
*dib* f	FW	ACAGGAAGAGCCACCTCACC
	RV	ACCATTCGGGTCCATTTGTT

*Results reported based on animals injected with dsRNA created using this primer set.

a [63].

b [59].

c [61].

d [71].

e [40].

f [35].

### Cloning and dsRNA synthesis

The PCR product was subsequently cloned into the TOPO-TA cloning vector (Life Technologies, Carlsbad, CA), and cells were transformed with this plasmid. The purified plasmid DNA was sequenced to confirm the identity of the cloned gene and the accuracy of the insertion into the TOPO vector. The plasmid DNA was linearized using *Spe1* and *Not1* restriction enzymes (New England Biolabs, Ipswich, MA) and subsequently used for single-stranded RNA (ssRNA) synthesis. ssRNA was synthesized using MEGAscript T3 and T7 kits (Life Technologies, Carlsbad, CA), according to the manufacturer's instructions. The complementary ssRNA were then combined and annealed to form a 2 µg/µl dsRNA solution using a standard annealing protocol [83]. The final annealed product was analyzed via gel electrophoresis to ensure proper annealing.

### dsRNA injection and treatment

To characterize the role of *vvl*, day 0 fifth instar *Tribolium* larvae were injected with dsRNA. Using a pulled 10 µl glass capillary needle connected to a syringe, dsRNA was manually injected into each animal. dsRNA was injected until the abdomen began to stretch (approximately 0.25 µl). Controls were injected with the similar volume of bacterial *amp^r^* dsRNA. Animals were also injected with *Met* dsRNA as a comparison to the *vvl* knockdown animals.

To determine the effect of JH on *vvl* knockdown animals, a subset of the injected larvae was also topically treated with 0.5 µl JH analog methoprene (5 ng or 15 µg) (Sigma-Aldrich, St. Louis, MO) dissolved in acetone. The same amount of acetone was applied to control larvae. All solutions were applied to the dorsal side of the animals immediately following injection with dsRNA (day 0 fifth instar) to mimic a constant level of JH. All experimentally treated animals were maintained at 29°C and 50% humidity. The animals were examined every other day and characterized in comparison to *amp^r^* dsRNA-injected animals treated with acetone. Initiation of metamorphosis was identified by observation of the characteristic J-shape (“J-hangers”) of the larva, immobile legs and the position of the larval eyes, which begin to migrate towards the posterior of the head segment.

### 20E and RH-2845 treatments

To determine the effect of *vvl* knockdown on ecdysone sensitivity, larvae injected with either *vvl* or *amp^r^* dsRNA as day 0 fifth instar larvae were treated with either 20E or an ecdysteroid mimic, RH-2485 methoxyfenozide (Sigma-Aldrich, St. Louis, MO) on day 2 of the fifth instar. For 20E, larvae were injected with 0.3 µl of 5 µg/µl, 0.5 µg/µl or 0.05 µg/µl 20E dissolved in water. Water injection was used as a control. For RH-2485, 0.5 µl of 0.2 or 2 µg/µl RH-2485 in acetone was topically applied to the larva. The latter amount of RH-2485 has previously been shown to activate ecdysone response genes in adult *Tribolium* [70]. Acetone was used as a control. Treated larvae were checked daily for signs of molting, prepupal development or death.

### Ecdysone quantification assay

To determine the effect of *vvl* knockdown on ecdysone titer, day 0 fifth instar larvae were injected with *amp^r^* or *vvl* dsRNA. On day 4, six larvae were collected and frozen at −80°C. Pooled larvae were homogenized in 100% methanol, centrifuged, and the supernatant was dried, then resuspended in 35 µl Grace's media. Triplicate 10 µl samples were assayed for ecdysteroids, and averaged. The radioimmunoassay was conducted as previously described using an antibody generously provided by Dr. Lawrence Gilbert that cross-reacted equivalently with ecdysone and 20-hydroxyecdysone [84]. Ecdysone was used as a standard.

### Quantitative RT-PCR

To determine the developmental expression profile of *vvl*, RNA was isolated from the whole body of sixth and seventh instar GA-1 strain larvae, and prepupae. Total RNA was isolated from the whole body of day 4 fifth instar larvae using Trizol extraction and treated with DNAse to remove genomic DNA. cDNA was synthesized from 1 µg of RNA as described above. Each biological sample consisted of pooled RNA from five fifth instar larvae or three seventh instar larvae or prepupae. Three biological replicates of each treatment were prepared, and SsoAdvanced SYBR Green Supermix (Bio-Rad Laboratories, Hercules, CA) was used for qPCR analyses.

To assess the effect of *vvl* on the expression of *E75*, *HR3*, *kr-h1*, *Met* and *jhamt3*, day 0 fifth instar GA-1 strain larvae were injected with *vvl* dsRNA or *amp^r^* dsRNA, and their RNA was isolated. A subset of larvae was also treated with 0.5 µl of methoprene (30 µg/µl) or acetone. The effects of *vvl* or *Met* knockdown on *Met* and *vvl* expression, respectively, were also assayed during the prepupal stage. For this, two animals injected with either *vvl*, *Met* or *amp^r^*dsRNA were pooled per biological sample, and three biological replicates were created.

The effect of *vvl* on the expression of ecdysone biosynthesis genes *spo*, *phm*, *shd*, *dib* and *sad*, was assayed by injecting day 0 fifth instar larvae with *vvl* dsRNA or *amp^r^* dsRNA, and splitting the larvae into the anterior (containing the thorax) and posterior (containing the abdomen) halves. RNA was pooled from five animals per biological sample, and three biological samples were prepared as described above. In addition, to determine whether *vvl* knockdown influences *spo* expression in the oenocytes, day 0 fifth instar KT817 strain larvae were injected with either *vvl* or *amp^r^* dsRNA. Oenocytes were visualized by GFP and isolated from day 4 fifth instar larvae. RNA was pooled from 15 larvae per treatment.

To determine the tissue specific expression of *vvl*, CNS with the associated structures including the CA, the fat body, the gut and the epidermis were isolated from 20 day 0 seventh instar GA-1 strain larvae. The tissues were pooled and RNA was extracted as described above. cDNA was created from 1 µg RNA.

To determine the effect of 20E on *HR3* expression in *vvl* dsRNA-injected animals, day 0 fifth instar GA-1 strain larvae were injected with *vvl* dsRNA. Two days later, larvae were injected with either 0.3 µl of water as a control or 0.3 µl of 0.5 µg/µl 20E. After 6 hrs, larvae were harvested for RNA isolation and subsequent cDNA synthesis as described above. Three larvae were pooled per biological replicate and three biological replicates were created. Each sample was run in triplicate with no-template controls. *rp49* was used as an internal control for all qPCRs.

### Semi-quantitative RT-PCR

In order to perform semi-quantitative RT-PCR, early prepupae were collected. Last instars injected with *amp^r^*, *vvl*, or *Met* dsRNA were collected as they prepupated. Three animals for each treatment were pooled in Trizol and prepared for total RNA isolation, and cDNA was synthesized from 1 µg of RNA as described above. PCR products were run on a gel and visualized using a UV-illuminator.

### *In situ* hybridization and visualization of GFP in KT817 strain

To create the probes, *vvl* gene was cloned into a TOPO-TA cloning vector and restriction digested as described above. The purified linearized plasmid DNA was used to create probes as described previously [85]. To examine the expression of *vvl* in GA-1 strain embryos, day 1 eggs were collected and dechorionated in 25% bleach. After heptane/formaldehyde fixation, eggs were cracked in heptane/methanol. Embryos were individually dissected out of the egg shell. Standard *in situ* hybridization protocol was followed. The sense probe was used as a control. To visualize GFP expression in KT817 embryos, eggs were dechorionated and fixed as above. Embryos were manually dissected out and mounted in 70% glycerol in PBS.

## Supporting Information

Figure S1 Phylogenetic analysis of POU domain proteins found on Genbank. A built-in Jukes-Cantor method was used on Geneious R7. The Genbank accession numbers are as follows: BmAcj6 (BAL03077); BmPOU6F2 (XP_004924143); BmPOUM1 (Q17237); BmPOUM2 (AAP93140); DmAcj6 (ABI34196); DmNubbin (P31368); DmPdm2 (ACQ45346); DmPdm3 (NP_610377); DmVvl (AAF50641); TcAcj6 (EFA01306); TcNubbin (XP_968439); TcPOU6F2 (EFA13130); TcVvl (EFA04675).
(TIF)

Figure S2 Amino acid alignment of Vvl and other POU domain factors. Amino acid sequence alignment for the conserved regions of Vvl in *Tribolium castaneum* and *Drosophila melanogaster*, POU-M2 in *Bombyx mori*, and POU3F4 in *Mus musculus*, *Xenopus laevis* and *Homo sapiens*.
(EPS)

Figure S3 Vvl knockdown using a second primer pair also results in lack of molting and precocious metamorphosis. (A) Prepupae formed after *amp^r^* (left) and *vvl* (right) dsRNA injection as day 0 fifth instar larvae. (B,C) Close-up of compound eyes (arrow) (B) and gin traps (arrowheads) (C) developing underneath the larval cuticle of *vvl* knockdown larvae. Knockdown of *vvl* was performed using dsRNA synthesized by using the following primer pairs: FW primer: TACCTTCCGGCGAGCAGT; RV primer: CCATGTGGTGGTTGTGATG.
(TIF)

Figure S4 Vvl binds to EcR and Usp, but not to Met. (A,B) GST-pulldown assay between GST-Vvl fusion protein and Flag-Met, Flag-Usp, Flag EcRA and Flag-EcRB fusion proteins. (A) Binding assay between Vvl and Met or Usp. Shown on the left are the 5% inputs of Flag-Usp and Flag-Met. Gel on the right shows the result after binding assay with glutathione sepharose-GST-Vvl fusion protein complex with either Flag-Usp or Flag-Met. (B) Binding assay between Vvl, EcRA, EcRB and Usp. (Lanes 1–3) the 10% inputs of Flag-EcRA, Flag-EcRB and Flag-Usp. (Lanes 4–6) The result after binding assay with glutathione sepharose-GST-Vvl fusion protein complex with either Flag-EcRA, Flag-EcRB or Flag-Usp. (Lanes 7 and 8) The result after binding assay with glutathione sepharose-GST-Vvl fusion protein complex with Flag-EcRA or Flag-EcRB and Flag-Usp. The entire open reading frames (ORFs) of *Tribolium vvl*, *Met*, two isoforms of *EcR* and *usp* cDNAs were isolated by RT-PCR. To amplify *Met*, *EcR* and *usp*, forward primers were designed for gene specific sequence with a Flag-tagged sequence (ATGGACTACAAAGACGATGACGACAAG) on the 5′-end. Amplified PCR products were cloned into pENTR vector using pENTR Directional TOPO Cloning Kits (Life Technologies, Carlsbad, CA). After sequencing, the *vvl* ORF was transferred into pDEST15, and Flag-tagged *Met*, *EcR* and *usp* were transferred into pDEST 42 using Gateway LR Clonase II Enzyme Mix (Life Technologies, Carlsbad, CA). The GST-Vvl fusion protein was purified by Glutathione Sepharose 4B (GE Healthcare, Little Chalfont, UK). Glutathione Sepharose-GST-Vvl fusion protein complexes and Flag-Met, Flag-EcRA, Flag-EcRB and/or Flag-Usp were incubated for 2 hours at 4°C for the binding assay. Flag-tagged protein was detected by 1∶1000 Anti-Flag M2 Monoclonal Antibody (Sigma-Aldrich, St. Louis, MO) followed by HRP conjugated goat anti-mouse IgG.
(TIF)
