# Supplementary figures and images for: The POU Factor Ventral Veins Lacking/Drifter Directs the Timing of Metamorphosis through Ecdysteroid and Juvenile Hormone Signaling
Source: PLoS Genet. 2014 Jun 19;10(6):e1004425. doi: 10.1371/journal.pgen.1004425 (PMC4063743; doi:10.1371/journal.pgen.1004425)

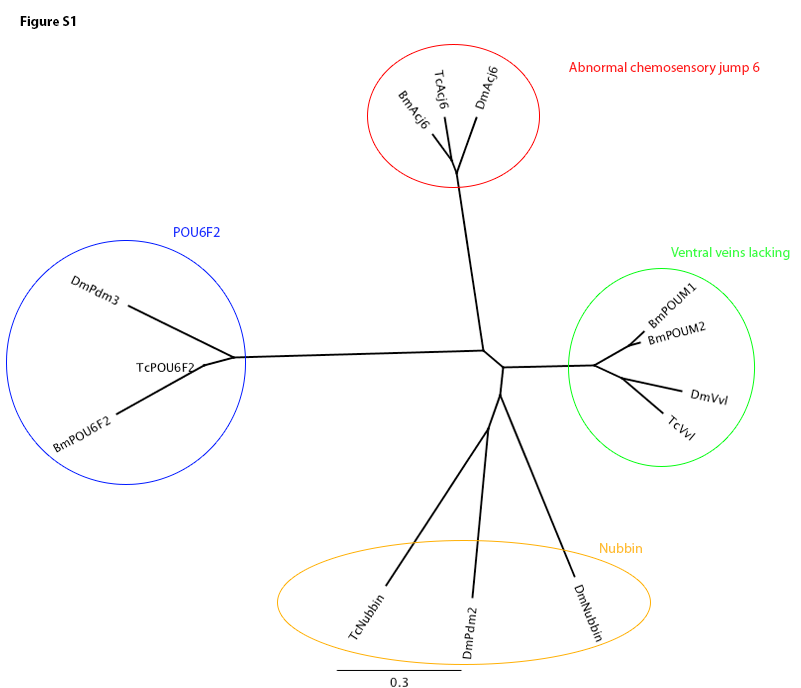

Supplement: Figure S1 — Phylogenetic analysis of POU domain proteins found on Genbank. A built-in Jukes-Cantor method was used on Geneious R7. The Genbank accession numbers are as follows: BmAcj6 (BAL03077); BmPOU6F2 (XP_004924143); BmPOUM1 (Q17237); BmPOUM2 (AAP93140); DmAcj6 (ABI34196); DmNubbin (P31368); DmPdm2 (ACQ45346); DmPdm3 (NP_610377); DmVvl (AAF50641); TcAcj6 (EFA01306); TcNubbin (XP_968439); TcPOU6F2 (EFA13130); TcVvl (EFA04675). (TIF) [file pgen.1004425.s001.tif]

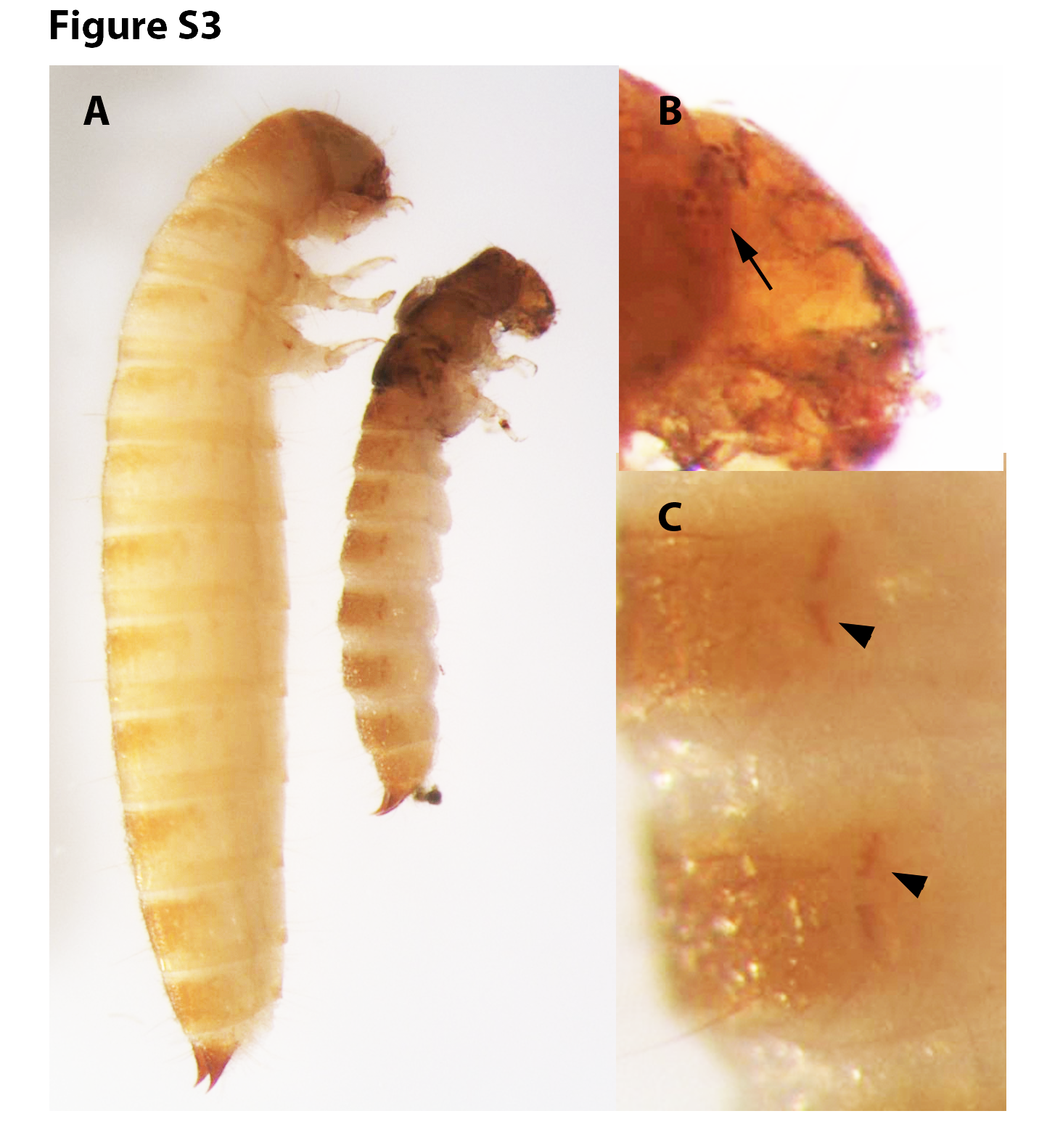

Supplement: Figure S3 — Vvl knockdown using a second primer pair also results in lack of molting and precocious metamorphosis. (A) Prepupae formed after ampr (left) and vvl (right) dsRNA injection as day 0 fifth instar larvae. (B,C) Close-up of compound eyes (arrow) (B) and gin traps (arrowheads) (C) developing underneath the larval cuticle of vvl knockdown larvae. Knockdown of vvl was performed using dsRNA synthesized by using the following primer pairs: FW primer: TACCTTCCGGCGAGCAGT; RV primer: CCATGTGGTGGTTGTGATG. (TIF) [file pgen.1004425.s003.tif]

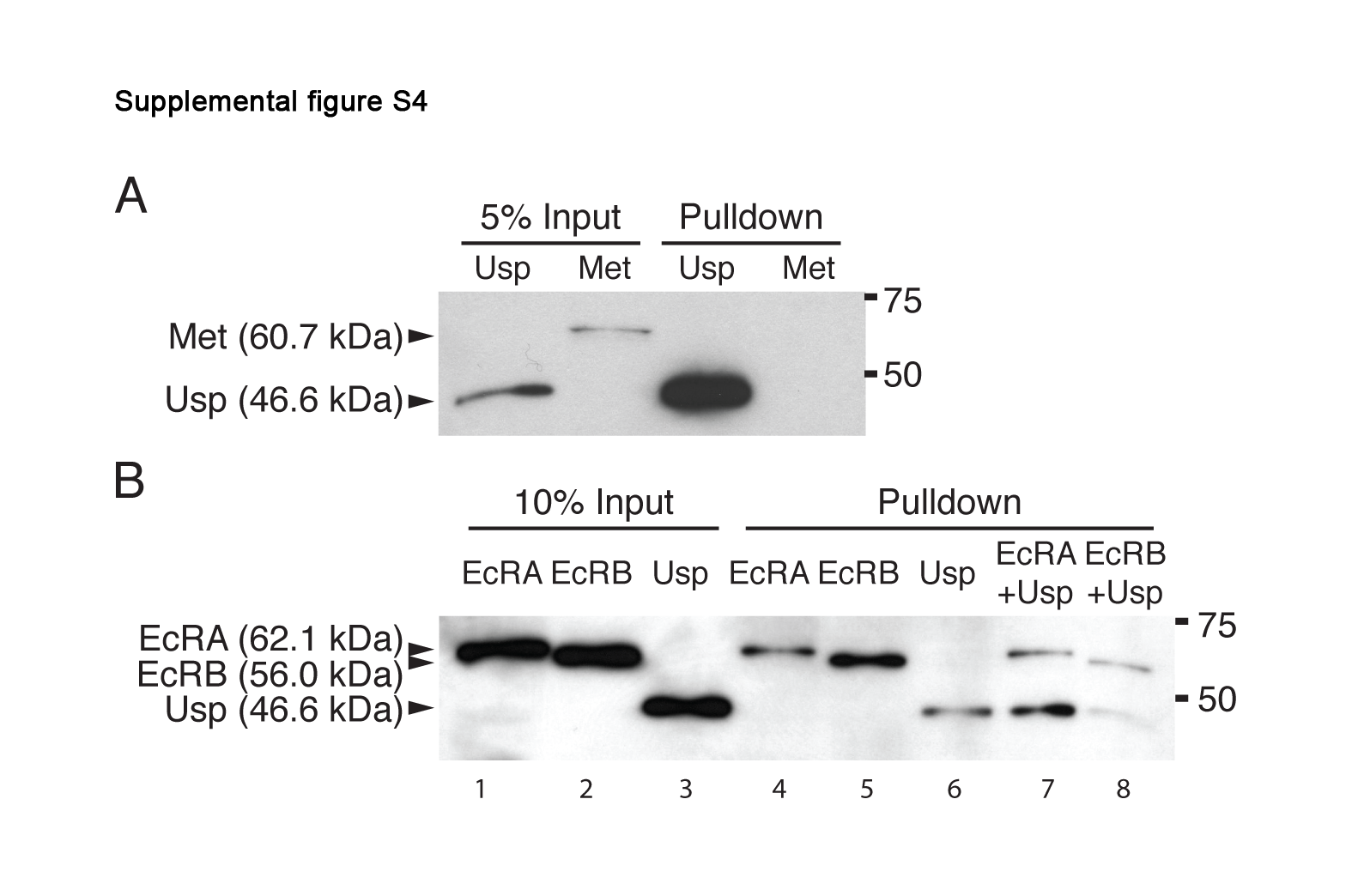

Supplement: Figure S4 — Vvl binds to EcR and Usp, but not to Met. (A,B) GST-pulldown assay between GST-Vvl fusion protein and Flag-Met, Flag-Usp, Flag EcRA and Flag-EcRB fusion proteins. (A) Binding assay between Vvl and Met or Usp. Shown on the left are the 5% inputs of Flag-Usp and Flag-Met. Gel on the right shows the result after binding assay with glutathione sepharose-GST-Vvl fusion protein complex with either Flag-Usp or Flag-Met. (B) Binding assay between Vvl, EcRA, EcRB and Usp. (Lanes 1–3) the 10% inputs of Flag-EcRA, Flag-EcRB and Flag-Usp. (Lanes 4–6) The result after binding assay with glutathione sepharose-GST-Vvl fusion protein complex with either Flag-EcRA, Flag-EcRB or Flag-Usp. (Lanes 7 and 8) The result after binding assay with glutathione sepharose-GST-Vvl fusion protein complex with Flag-EcRA or Flag-EcRB and Flag-Usp. The entire open reading frames (ORFs) of Tribolium vvl, Met, two isoforms of EcR and usp cDNAs were isolated by RT-PCR. To amplify Met, EcR and usp, forward primers were designed for gene specific sequence with a Flag-tagged sequence (ATGGACTACAAAGACGATGACGACAAG) on the 5′-end. Amplified PCR products were cloned into pENTR vector using pENTR Directional TOPO Cloning Kits (Life Technologies, Carlsbad, CA). After sequencing, the vvl ORF was transferred into pDEST15, and Flag-tagged Met, EcR and usp were transferred into pDEST 42 using Gateway LR Clonase II Enzyme Mix (Life Technologies, Carlsbad, CA). The GST-Vvl fusion protein was purified by Glutathione Sepharose 4B (GE Healthcare, Little Chalfont, UK). Glutathione Sepharose-GST-Vvl fusion protein complexes and Flag-Met, Flag-EcRA, Flag-EcRB and/or Flag-Usp were incubated for 2 hours at 4°C for the binding assay. Flag-tagged protein was detected by 1∶1000 Anti-Flag M2 Monoclonal Antibody (Sigma-Aldrich, St. Louis, MO) followed by HRP conjugated goat anti-mouse IgG. (TIF) [file pgen.1004425.s004.tif]
